# Prevalence of *Escherichia coli* Virulence Genes in Patients with Diarrhea and a Subpopulation of Healthy Volunteers in Madrid, Spain

**DOI:** 10.3389/fmicb.2016.00641

**Published:** 2016-05-02

**Authors:** Adriana Cabal, María García-Castillo, Rafael Cantón, Christian Gortázar, Lucas Domínguez, Julio Álvarez

**Affiliations:** ^1^VISAVET Health Surveillance Centre, Universidad ComplutenseMadrid, Spain; ^2^SaBio-IREC (CSIC-UCLM-JCCM)Ciudad Real, Spain; ^3^Servicio de Microbiología, Hospital Universitario Ramón y CajalMadrid, Spain; ^4^Servicio de Microbiología, Instituto Ramón y Cajal de Investigación SanitariaMadrid, Spain; ^5^Department of Veterinary Population Medicine, College of Veterinary Medicine, University of Minnesota, St. PaulMN, USA

**Keywords:** *E. coli*, diarrhea, virulence genes, pathotypes, prevalence

## Abstract

Etiological diagnosis of diarrheal diseases may be complicated by their multi-factorial nature. In addition, *Escherichia coli* strains present in the gut can occasionally harbor virulence genes (VGs) without causing disease, which complicates the assessment of their clinical significance in particular. The aim of this study was to detect and quantify nine VGs (*stx*1, *stx*2, *eae, agg*R, *ehx*A, *inv*A, *est, elt* and *bfp*A) typically present in five *E. coli* enteric pathotypes [enterohaemorrhagic *E. coli* (EHEC), enterotoxigenic *E. coli* (ETEC), enteropathogenic *E. coli* (EPEC), enteroaggregative *E. coli* (EAEC), and enteroinvasive *E. coli* (EIEC)] in fecal samples collected from 49 patients with acute diarrhea and 32 healthy controls from Madrid, Spain. In addition, the presence of four serotype-related genes (*wzx*_O104_ and *fli*C_H4_, *rfb*_O157_, and *fli*C_H7_) was also determined. Presence of target genes was assessed using a quantitative real-time PCR assay previously developed, and the association of presence and burden of VGs with clinical disease and/or other risk factors was explored. Prevalence of *ehx*A [typically associated with Shigatoxin producing *E.* coli (STEC) and (EPEC), *invA* (EIEC), and the *rfb*_O157_+*fli*C_H7_ (STEC)] combination were significantly (*p* < 0.02) higher in the diarrheic group, while the *wzx*_O104_+*fli*C_H4_ combination was significantly (*p* = 0.014) more prevalent in the control group. On the other hand, *eae* was detected in more than 90% of the individuals in both patient and control populations, and it was not associated with *bfp*A, suggesting the absence of typical EPEC. No significant differences in the quantitative values were detected for any VG among study groups, but the difference in the load of *agg*R (EAEC) and *inv*A in the patients with respect to the controls was close to the significance, suggesting a potential role of these VGs in the clinical signs observed when they are present at high levels.

## Introduction

*Escherichia coli* are commensal bacteria living in the intestinal tract of animals and humans. Some innocuous strains can incorporate virulence genes (VGs) by lateral gene transfer that may allow them to cause intestinal and extraintestinal disease (Ochman et al., 2000). There are six pathotypes than can produce intestinal disease in humans: STEC (Shigatoxin-producing *E. coli*, including EHEC – enterohaemorrhagic *E. coli*), EPEC (Enteropathogenic *E. coli*), ETEC (Enterotoxigenic *E. coli*), EIEC (Enteroinvasive *E. coli*), EAEC (Enteroaggregative *E. coli*), and DAEC (Diffusely adherent *E. coli*; Kaper, 2005). *E. coli* strains are classified into these pathotypes depending on the presence/absence of several combinations of VGs. In EPEC, the virulence machinery is based on the carriage of *eae, tir*, and other proteins required for causing attachment and effacement (A/E) lesions and they are encoded on a chromosomal pathogenicity island known as the locus for enterocyte effacement (LEE; Kaper et al., 2004). Typical EPEC (tEPEC) strains also possess the EAF plasmid, which encodes for the bundle-forming pili (*bfp*A gene) and the *per*ABC genes that regulate *eae* expression. Atypical EPEC (aEPEC) strains lack the EAF plasmid (Johnson and Nolan, 2009), and can affect children in both developed and developing countries, and together with EHEC and EAEC strains, are considered an emerging pathogen (Trabulsi et al., 2002; Huang et al., 2004). EAEC strains possess a p*AA* plasmid for fimbriae production, which contains the *agg*R transcriptional activator and its regulated genes (Kaper et al., 2004; Johnson and Nolan, 2009). In addition, there is a cluster of atypical EAEC strains that lacks *agg*R and may not produce diarrhea in humans. EHEC infection can lead to hemorrhagic colitis (HC) and in the most severe cases hemolytic-uremic syndrome (HUS; Paton and Paton, 1998), which is often associated with serotypes O157:H7 and, recently, O104:H4. Their virulence is mainly due to two types of Shiga-like toxins, *Stx*1 and *Stx*2, but also to *eae* (intimin) and *ehx*A (enterohaemolysin). EIEC carries an invasion plasmid (p*Inv*) which encodes the *vir* regulon, which is key for intestinal dysentery. ETEC has been reported as well as the causative agent of the traveler’s diarrhea in patients who traveled to developing countries (Gascon et al., 1998). ETEC pathogenicity is mainly determined by the production of heat-stable (ST) and labile-stable (LT) enterotoxins. In patients with an EIEC or ETEC infection, symptoms usually can be resolved without any complication while in severe cases of EHEC, 10% of the patients can develop HUS (Nataro and Kaper, 1998). New pathotypes may also emerge due to the genetic recombination of some of these VGs, as demonstrated in the 2011 outbreak in Germany caused by a new O104:H4 EAEC/EHEC strain that affected 3,816 people and caused 54 deaths (Frank et al., 2011). In Spain, only one HUS STEC case and one non-HUS case due to the infection with the EAEC/EHEC strain were reported (Mora et al., 2011).

In both developing and developed countries, the causative agents of diarrhea may vary. In developed countries, diarrhea caused by *E. coli* (attributed to EPEC, ETEC, and other pathotypes) in children accounts for less than 0.4% of the cases (Fletcher et al., 2013) while the detection rate of *E. coli* in adults (>12 years) is even lower. In Spain, EPEC, STEC, and EAEC are the *E. coli* pathotypes most commonly isolated in clinical samples (Blanco et al., 2006). However, there is limited knowledge about the occurrence and quantitative burden of *E. coli* VGs in healthy individuals, which could help to evaluate results from clinically affected individuals.

The objective of this study was to evaluate if there were differences in the presentation and amount of VGs in feces from patients with diarrhea and a subpopulation of healthy individuals potentially exposed to certain *E. coli* pathotypes (veterinary students and laboratory staff) using a direct quantitative real-time PCR. Four serotype-related genes associated with the O157:H7 and O104:H4 serotypes (previously associated with outbreaks of importance in public health) were also investigated by qPCR. Additionally, we evaluated the association between certain individual characteristics and the presence and burden of VGs in the healthy population to identify factors promoting exposure.

## Materials and Methods

### Study Population

Feces from a total of 81 individuals from two different populations were collected. First, 49 samples from individuals belonging to a clinically affected population (“cases”) were selected randomly among those received during a 1-month period at the Ramón y Cajal Hospital (Madrid, Spain) from patients suffering from acute diarrhea (aqueous to mucoid) of an unknown origin. Information on the gender and age of the patients and the origin of the patient (hospitalization area, emergency room or primary health center) were recovered from the clinical chart from all but three patients. Samples from 30 female and 16 male patients were included in the study, with ages ranging from 1 to 94 years old. Thirty-three went to their Primary Health Center, 10 went directly to the Hospital and 3 were admitted in the Emergency room. None of the patients had received antimicrobial treatment before their samples were collected.

The healthy population (“controls”) consisted of 20 veterinary students at the University Complutense and 12 members of the VISAVET Health Surveillance Center staff (20 females and 12 males with ages ranging between 18 and 44 years) that volunteered to participate in the project. None of them presented any gastrointestinal symptoms when the samples were collected. Information on the gender, eating habits, traveling history, previous intestinal illnesses, and antimicrobial therapy were collected using a questionnaire (available upon request). This study was authorized by the Ethics Committee for Clinical Research from the Hospital Ramon y Cajal (Reference 098/12).

### VG Detection

Gene selection was made based on a previous study (Cabal et al., 2013) so that it included the most representative VGs from the intestinal pathotypes of *E. coli* and four genes associated with relevant serotypes for public health (Supplementary Table S1).

Three grams of the fecal samples were mixed individually with 3 ml of PBS (1/2 dilution) and homogenized vigorously. Four-hundred milligrams of the resulting solution were used for DNA extraction, which was performed using a commercial kit (Qiagen DNA stool mini kit) according to the manufacturer’s instructions. Then, the amplification of the targets was performed using a real-time PCR assay (qPCR) as previously described (Cabal et al., 2015). Briefly, for detection of VGs, a conventional PCR was first performed using specific primers (Supplementary Table S2) for control strains (see below) to obtain specific PCR products, which were ten-fold diluted in order to generate the standard curves. For the serotype related genes, 10-fold dilutions of STEC control strains were performed and further used for building the standards needed for the qPCR. Then, qPCR analysis of the clinical samples was carried out using primers and probes described in Supplementary Tables S1 and S3, and quantification was achieved using the standard curves generated as explained below. The final gene copy number per milligram of sample was estimated taking into account the initial weight of the stool, the dilution factor and the DNA volume added to the qPCR.

The following *E. coli* control strains were included in all qPCR reactions as positive controls and were also used to generate the standard curves: STEC O157:H7 (strain CNM 2686/03 positive to *stx*1, sxt2, *eae*, and *ehx*A), a typical EPEC (strain CNM764, positive to *bfp*A, and *eae*), provided by Dr. Silvia Herrera León (National Center of Microbiology, Institute of Health Carlos III, Madrid, Spain), ETEC (strain H10407, positive to *elt*, and *est*), one EIEC (strain 1280 positive to *inv*A), and the EAEC/EHEC outbreak strain (LB226692 positive to *agg*R and *stx*2), provided by Dr. Martina Bielaszewska (Institute of Hygiene, Munster University, Münster, Germany).

### Statistical Analysis

The proportion of positive samples and the quantitative results for each gene in the case and control groups were compared using Pearson’s and Fisher’s Exact χ^2^ tests and Mann–Whitney tests, respectively. In addition, results of the PCR analysis among the controls were also compared depending on their individual characteristics. Calculations were performed using SPSS V20.0 (IBM, Chicago, IL, USA).

## Results

Qualitative results obtained in case and control samples are depicted in **Table 1**. The most prevalent VGs among patients were *eae* (*n* = 45; 91.8%), *agg*R (*n* = 31; 63.3%), and *inv*A (*n* = 40; 81.6%), while *eae* (*n* = 30; 93.8%), and *agg*R (*n* = 16; 50%) were the most commonly found VGs in the controls (**Table 1**). *Ehx*A and *bfp*A were both moderately represented in patients (*n* = 20; 40.8% and *n* = 14; 28.6%) and controls (*n* = 5; 15.6% and *n* = 10; 31.3%), respectively. *Stx*1 was infrequent in both groups (*n* = 2; 4.1% in patients and *n* = 1; 3.1% in controls). *Stx*2 was only detected in controls (*n* = 1, 3.1%) while *est* was only found in patients (*n* = 1; 2%). Interestingly, the *elt* gene was not found in any case or control sample. *Inv*A, *ehx*A, and the simultaneous presence of *rfb*_O157_ with *fli*C_H7_ were significantly more prevalent (*p* < 0.05) in the diarrheal patients than in healthy controls, while the opposite was true for the simultaneous detection of both *wzx*_O104_ and *fli*C_H4_ (**Table 1**).

**Table 1:** Percentage and 95% confidence intervals (CI) of the proportion of samples positive to each molecular target in patients (cases) and healthy volunteers (controls).

Molecular target	Cases (*n* = 49)	Controls (*n* = 32)	*p*
*stx*1	4.1 (1.1-13.7)	3.1 (0.55-15.7)	0.66
*stx*2	0.0 (0.0-7.27)	3.1 (0.55-15.7)	0.40
*eae*	91.8 (80.8-96.8)	93.8 (79.9-98.3)	0.56
*ehx*A	40.8 (28.2-54.8)	15.6 (6.86-31.8)	**0.014**
*elt*	0.0 (0.0-7.27)	0 (0.0-10.7)	–
*est*	2.04 (0.36-10.7)	0 (0.0-10.7)	0.61
*inv*A	81.6 (68.6-90.0)	6.25 (1.73-20.1)	**<0.001**
*bfp*A	28.6 (17.8-42.4)	31.3 (18.0-48.6)	0.49
*agg*R	63.3 (49.3-75.3)	50.0 (33.6-66.4)	0.17
O157+H7^a^	53.1 (39.4-66.3)	18.8 (8.89-35.3)	**0.002**
O104+H4^a^	2.04 (0.36-10.7)	18.8 (8.89-35.3)	**0.014**

*^a^Indicates simultaneous detection in a given sample of genes linked to the somatic and flagellar antigens (*rfb*_O157_, *fli*C_H7_, and *wzx*_O104_, *fli*C_H4_).*

*Significant p-values are highlighted in bold.*

No significant differences between age or sex and detection of VGs were detected in any of the two groups. Analysis of the information collected in the survey of the control population revealed that a significantly higher proportion of this group was positive for the *rfb*_O157_ gene among those taking antibiotics in comparison with those who did not (*p* = 0.01). Marginally significant differences between the presence of *ehx*A and the consumption of antibiotics was also observed (higher in those taking antibiotics, *p* = 0.057). Also, proportion of controls positive to *agg*R and *bfp*A was marginally significantly higher (*p* = 0.1 and *p* = 0.08, respectively) among those with travel history (**Table 2**). No significant differences in the proportion of positive samples to any other VG and the variables under study were detected.

**Table 2:** Characteristics of the control population and proportion of positive samples to the virulence genes (VGs) for which marginally significant (*p* < 0.1) differences were found.

Variable	Response	*N* (%)	*ehx*A^a^	*rfb*_O157_^a^	*bfp*A^a^	*agg*R^a^
Received antibiotic treatment in the last 3 months	Yes	7 (21, 9%)	42, 8%	71, 4%		
	No	25 (78, 1%)	8%	16%		
Foreign travel in the last 3 months	Yes	4 (12,5%)			75%	100%
	No	28 (87, 5%)			25%	42,8%
Gastrointestinal problems in the last 3 months	Yes	11 (34, 4%)				
	No	21 (65, 6%)				
Frequent contact with animals	Yes	27 (84, 4%)				
	No	5 (15, 6%)				
Vegetarian	Yes	1 (3, 1%)				
	No	31 (96, 9%)				
Regular consumption of probiotics	Yes	23 (71, 9%)				
	No	9 (28, 1%)				

*^a^Percentage of positive samples among the controls that were positive to each virulence factor.*

In the quantitative analysis, only VGs that were present in at least five samples (*ehx*A, *eae, agg*R, *bfp*A, *rfb*_O157_, *fli*C_H7_, and *fli*C_H4_) were considered (**Table 3**). Within these analyses, no significant differences between the quantitative values found in each group were detected, except for the *fli*C_H4_ gene [with a significantly (*p* < 0.007) higher number of gene copies per milligram of sample in the control group]. Marginally significant differences were found for *agg*R and *inv*A, present at higher quantities in the patients (*p* = 0.07 and 0.084, respectively). In healthy controls, the maximum gene copy number for a given VG per milligram of feces was always under 10e^+04^. In contrast, for the patients, there was a maximum of 10e^+07^ gene copy number. If a tentative cut-off value was established so that patients with gene copy numbers per milligram of feces >10e^+03^ (Cabal et al., 2015) were considered positive for that VG, only four patients would be positive for VGs from pathogenic *E. coli* compared to one control. Those four patients with copy numbers above 10e^+03^ for at least one VG were positive to *eae*/*agg*R/*est, agg*R/*inv*A, *eae*/*bfp*A, and *eae*, respectively.

**Table 3:** Quantitative values (median) for VGs present in at least five samples in patients and controls.

	Patients		Controls		*p*-Value
Gene	Median values (*n*)^a^	Samples > 1.00e^+03^ (*n* = 5)^b^	Median values (*n*)^a^	Samples > 1.00e^+03^ (*n* = 5)^b^	
*eae*	0.82 (45)	4	0.59 (30)	1	0.88
*ehxA*	0.22 (20)	0	0.24 (5)	0	0.92
*agg*R	1.41 (31)	2	0.87 (16)	0	0.07
*bfp*A	1.72 (14)	1	4.56 (10)	0	0.62
*inv*A	43.5 (40)	1	8.04 (2)	0	0.084
*rfb*_O157_	0.50 (30)	0	0.84 (9)	0	0.27
*fli*C_H7_	1.21 (42)	0	3.06 (16)	0	0.13
*fli*C_H4_	182.4 (29)	0	14.760 (23)	0	0.007

*^a^Median values are expressed in gene copies per milligram of feces. ^b^Number of positive individuals when gene copy number per milligram of feces is equal or above 1.00e^*+03*^.*

## Discussion

In this study, we estimated the qualitative and quantitative (gene copy number per milligram of sample) abundance of VGs in the feces of two groups of individuals, healthy volunteers and patients with diarrhea. Overall, the prevalence of VGs in both groups was high. In fact, while the presence of VGs in the feces of patients with diarrhea was expected to some extent, healthy controls carried VGs as well (Stephan et al., 2000; Fujihara et al., 2009). Humans have been proven to act sometimes as mere carriers of these VGs without being necessarily related with intestinal disease (Jenkins et al., 2007) since they may need to coexist with other VGs and eventually in an adequate microorganisms to produce clinical symptoms (Rosenshine et al., 1996; Barletta et al., 2011). The high prevalence observed may be due in part to the analytical approach adopted here, since qPCR-based direct detection of DNA extracted from fecal samples may yield a higher analytic sensitivity than isolation of *E. coli* followed by PCR-detection.

The *agg*R gene was observed at high prevalence in both patients (63.3%) and controls (50%), in contrast with a similar study by Samie et al. (2007) that described higher prevalence of *agg*R in the patient group. However, the higher numbers of gene copies in positive case samples compared to the controls, which was close to the statistical significance (**Table 3**), was in line with the findings of that study, suggesting that that *agg*R may contribute to the patient symptoms (Samie et al., 2007).

The *wzx*_O104_/*fli*C_H4_ combination, recently associated with EAEC (Bielaszewska et al., 2011), was more common in healthy individuals (18.8%) than in patients (2.04%), with most of these positive control samples being positive also to *agg*R (4/6 and 66.7%), suggesting asymptomatic carriage of EAEC and/or EAEC/EHEC as previously reported (Mathewson et al., 1985; Balabanova et al., 2013).

In contrast, the prevalence and quantities of *bfp*A, which may indicate presence of tEPEC, was similar in both patients and healthy individuals, suggesting a limited pathogenic role as previously proposed (Wang et al., 2013). Interestingly, the marginally significant differences found in the controls who traveled abroad for *bfp*A but also for *agg*R may indicate that EPEC and EAEC are the pathotypes most probably acquired during international travels, as previously described (Tangden et al., 2010; Laaveri et al., 2014). Detection of other tEPEC virulence markers in *bfp*A-positive samples could help in elucidating the real role of *bfp*A in disease pathogenesis.

In the case of *eae*, its large prevalence (>90%) in *bfp*A-negative fecal samples could indicate the presence of aEPEC. In addition, the fact that *eae* frequencies were similar in both groups was in agreement with previous studies (Fujihara et al., 2009; Ghosh and Ali, 2010; Schmidt, 2010), and therefore it also questions its potential pathogenic role in our study population.

The low prevalence found for *stx*-genes was in agreement with that reported by Pradel et al. (2000), which was also similar for both patients and controls. Still, the high frequencies obtained for *eae, ehx*A, and *rfb*_O157_/*fli*C_H7_ genes (particularly among case samples) may indicate the presence of O157:H7 strains despite the low prevalence obtained for the Shiga toxins. It has been shown that both EPEC and EHEC O157:H7 strains can exist in patients with HUS or diarrhea (Ferdous et al., 2015), and a whole genome analysis approach would be needed to differentiate them.

In the case of EIEC, represented in this study by *inv*A, the high prevalence obtained for patients together with a higher gene copy number in this group could indicate that *inv*A is directly related with pathogenicity, thus explaining its absence (or presence at very low levels) in healthy controls (Labbé and García, 2013). Bruijnesteijn van Coppenraet et al. (2015) also found EIEC-typical genes in a significantly higher proportion of cases compared with a control population, while no differences were observed for STEC-associated genes.

Lastly, detection rates for *est* and/or *elt*, usually associated with the ETEC pathotype and therefore with Traveler’s diarrhea, were low among diarrheal patients, although interpretation of these results is not possible due to the lack of information on the patient’s travel history (Qadri et al., 2005; Rivera et al., 2013). Among the variables included in the survey performed in the control population, we only observed a significant association between *ehx*A or *rfb*O157 and antibiotic intake (**Table 2**). A marginally significant association between *agg*R or *bfp*A and foreign travel was seen in agreement with previous reports (Evans and Evans, 1996; Jiang et al., 2002).

Comparison of results obtained in the case and control populations obtained here must be performed carefully since, while cases were sampled randomly among those submitted to a large hospital in Madrid, our control population was severely biased toward a very specific subpopulation (veterinary students and laboratory staff) that could be exposed to certain risks (i.e., STEC of animal origin), and may thus not be representative of the general population. Interestingly, prevalence of VGs associated with STEC (i.e., *stx*1, and *stx*2) was very low in both cases and controls (**Table 1**), thus suggesting a non-differential degree of exposure to sources of this particular pathotype. Another limitation was the limited size of our sample; even though matching by age and sex could have helped to increase the power of the study, this information was not available when controls were recruited and was thus not possible.

In summary, our strategy allowed the assessment of the prevalence and abundance of VGs associated with diarrheagenic *E. coli* in healthy volunteers and also in patients with diarrhea. In some cases both groups were carriers of the same VGs, highlighting the fact that not all VGs might be linked with pathogenicity or may need additional association with other VG to produce diarrhea. Moreover, the VG quantification could help to determine whether a VG is crucial for the onset of the diarrhea or not and may facilitate the isolation of positive colonies. Although some individual characteristics were marginally associated with increased odds of being positive for certain VGs in the control population, the limited sample size used here prevents extracting definitive conclusions.

## Author Contributions

RC, CG, LD, and JÁ conceived and designed the study. AC and MG-C collected the samples and performed the laboratory analyses. AC and JÁ analyzed the data and drafted the first version of the manuscript. All authors revised the manuscript critically and contributed to the final version.

## Conflict of Interest Statement

The authors declare that the research was conducted in the absence of any commercial or financial relationships that could be construed as a potential conflict of interest.
