# Supplementary material for: Prevalence of Escherichia coli Virulence Genes in Patients with Diarrhea and a Subpopulation of Healthy Volunteers in Madrid, Spain
Source: Front Microbiol. 2016 May 2;7:641. doi: 10.3389/fmicb.2016.00641 (PMC4859089; doi:10.3389/fmicb.2016.00641)
Supplement: Supplementary file 1 [file Table_1.DOCX]

**Table S1.** Primers and PCR conditions used for each target (source: Cabal et al. 2013)

| **Target** | **Primers** | **Pathotype** | **T_a_(ºC)^a^** | **Amplicon (bp)** | **Oligonucleotide sequence (5´🡪 3')** |
| --- | --- | --- | --- | --- | --- |
| Shiga toxin 1 (*stx*1) | *stx*1-F | EHEC | 55 | 107 | GCAAAGAMGTATGTWGATTCG |
|  | *stx*1-R |  |  |  | GWGCCACTATCAATCATCAG |
| Shiga toxin 2 (*stx*2) | *stx*2-F | EHEC | 55 | 82 | AATGCAAATCAGTCGTCAC |
|  | *stx*2-R |  |  |  | TGCATCTCTGGTCATTGTAT |
|  | *stx*2-F var |  | 55 | 82 | GATGCAGATTRRKCGYCAT |
|  | *stx*2-R var |  |  |  | TGATGCGCGGGTCATGGAAC |
| Intimin | *eae-*F | EHEC/EPEC | 52 | 92 | GCTATAACRTCTTCATTGATC |
|  | *eae-*R |  |  |  | RCTACTTTTRAAATAGTCTCG |
| Enterohaemolysin (*ehx*A) | *ehx*A-F | EHEC/EPEC | 55 | 86 | GCACCACAACTTGAYAAACT |
|  | *ehx*A-R |  |  |  | CCAGATTATTACCTACATTYTCAG |
| ST toxin (*est*) | *est-*F | ETEC | 54 | 69/72 | TGAAAGCATGAATRGTAGCAA |
|  | *est-*R |  |  |  | TTAATAACATSSAGCACAGG |
|  | *est-*F var |  | 54 | 69/73 | TCAGAAAATATGAAYAACACATT |
|  | *est*-R var |  |  |  | TAATAGCACCCGGTACAAG |
| LT toxin (*elt*) | *elt*-F | ETEC | 54 | 142 | GGYAAAAGAGAAATGGTTAT |
|  | *elt*-R |  |  |  | TCTCGGTCAGATATGYGATTC |
| Bundle- forming pilus (*bfp*A) | *bfp*A-F | EPEC | 53 | 109 | CMGGTGTGATGTTTTACTAC |
|  | *bfp*A-R |  |  |  | TGCCCAATATACARACCAT |
| Invasion plasmid (*spa*24) | p*Inv-*F | EIEC | 53 | 159 | CCAATCACAATATCAGTACCA |
|  | p*Inv-*R |  |  |  | AAAGAGCCTTATTACCCATAT |
| Enteroaggregative regulator (*agg*R) | aggR-F | EAEC | 56 | 94 | TTTATCGCAATCAGATTAARC |
|  | aggR-R |  |  |  | GGACAACTRCAAGCATCTAC |
| *rbf*_O157_ | *rfb* _O157_-F | EHEC | 55 | 125 | CAAAAGGAAACTATATTCAGAAGT |
|  | *rfb* _O157_-R |  |  |  | CGATATACCTAACGCTAACAA |
| *fli*C_H7_ | *fli*C _H7_-F | EHEC | 55 | 91 | CGACAGGTCTTTATGATCTGA |
|  | *fli*C _H7_-R |  |  |  | ACTGTGACTTTATCGCCATT |
| *wzx*_O104_ | *wzx* _O104_-F | EAEC | 55 | 99 | GCGCAAAGAATTTCAACTT |
|  | *wzx* _O104_-R |  |  |  | TGTAAAATCCTTTAAACTATACG |
| *fli*C_H4_ | *fli*C _H4_-F | EAEC | 55 | 192 | CTGGGGGTAAACAAGTCAA |
|  | *fli*C _H4_-R |  |  |  | CCAGTGCTTTTAACGGATC |

^a^Annealing temperature
