# Supplementary material for: Prevalence of Escherichia coli Virulence Genes in Patients with Diarrhea and a Subpopulation of Healthy Volunteers in Madrid, Spain
Source: Front Microbiol. 2016 May 2;7:641. doi: 10.3389/fmicb.2016.00641 (PMC4859089; doi:10.3389/fmicb.2016.00641)
Supplement: Supplementary file 2 [file Table_2.DOCX]

**Table S2.** Primers used in the conventional PCR for generation of standard curves (source: Cabal et al. 2015).

| **Gene** | **Ta (ºC)^a^** | **Primer sequence 5´ --> 3´ U** | **Primer sequence 5´ --> 3´ L** |
| --- | --- | --- | --- |
| *stx*1 | 55 | GTTAATGTGGTKGCGAAGGA | ACGCTWCCCAGRATTGCAT |
|  |  | GTTAATGCGATTGCTAAGGA | --- |
| *stx*2 | 55 | GGYTTTTCTWCGGTATCCYATT | CGCHAT AAWYATCTTCTTCAT |
|  |  | ACTGTGGATTTCTCTTCGCA | --- |
|  |  | ACCTTAGAYTTCTCRACWGC | --- |
| *eae* | 52 | TTRAAAMCWGGTGAAACTGTT | GGCTGCCYGATAATGTTCTT |
| *ehx*A | 55 | CAGTTTTTTGGTRCAGCAGAA | CCTGCAATACCTTCACGTCAC |
| *agg*R | 56 | ACAAAAGAAGGAAGCAATACA | TGCTTTGCTCATTCTTGATT |
| *est* | 54 | AAACCAGATAGCCAGACAAT | TTATTATGATTTTCTCAGCACC |
| *elt* | 54 | TTTTATTAGCATCGCCATTAT | ACAGTTTAAGGATCGGTATTG |
| *inv*A | 53 | GTTTGCAGCAGGTTCCATC | CCAATAGWTAATCCAATAACCGT |
| *bfp*A | 53 | GAATAAGAAATACGARAAAGGTC | ACAGGGCGTATTATGTAGATTA |

^a^Annealing temperature
