# Supplementary material for: Prevalence of Escherichia coli Virulence Genes in Patients with Diarrhea and a Subpopulation of Healthy Volunteers in Madrid, Spain
Source: Front Microbiol. 2016 May 2;7:641. doi: 10.3389/fmicb.2016.00641 (PMC4859089; doi:10.3389/fmicb.2016.00641)
Supplement: Supplementary file 3 [file Table_3.DOCX]

**Table S3.** Probes used for each target in the qPCRs (source: Cabal et al. 2015).

| **Gene** | **Pathotype/ Antigen** | **Probe sequence 5´ --> 3´** | **Flurophore(5´)** | **Quencher (3´)** |
| --- | --- | --- | --- | --- |
| *stx*1 | STEC | TTCGCTCTGCAATAGGTACKCCAT | ROX | BHQ2 |
| *stx*2 | STEC | CACTGGTTTCATCATATCTGGCGTT | FAM | BHQ1 |
| *eae* | STEC/EPEC | TTCGCCACCAATACCTAAACGG | FAM | BHQ1 |
| *ehx*A | STEC/EPEC | TTTACTCCCAACGTTCTGATACTTCTG | FAM | BHQ1 |
| *agg*R | EAEC | ACATTAAGACGCCTAAAGGATGCC | ROX | BHQ2 |
| *est* | ETEC | CAGGATTACAACAMARTTCACAGCAGT | FAM | BHQ1 |
| *elt* | ETEC | TGTGTCCTTCATCCTTTCAATGGC | ROX | BHQ2 |
| *inv*A | EIEC | AGACACATTACCTCCATCATCTAAGCA | ROX | BHQ2 |
| *bfp*A | EPEC | AGTCTGCGTCTGATTCCAATAAGKC | FAM | BHQ1 |
| *rfb*_O157_ | O157 | ATTCCTCTCTTTCCTCTGCGGTC | FAM | BHQ1 |
| *fli*C_H7_ | H7 | CCTTGTTAACTACCGATGCTGCATT | ROX | BHQ2 |
| *wzx*_O104_ | O104 | TGAAATGACACCACTTATTGCTAATACA | ROX | BHQ2 |
| *fli*C_H4_ | H4 | TCTTACACTGACACCGCGTCTAACA | FAM | BHQ1 |
